# How can we strengthen the fragile relationship between families with mental health needs and children’s social care?

**DOI:** 10.12968/jfch.2025.2.4.190

**Published:** 2025-05-26

**Authors:** Katy Cleece, Parisa Quaynor, Carley Hall, Katalin Ujhelyi Gomez

**Affiliations:** 1 Social Work Research Lead, Lancashire and South Cumbria NHS Foundation Trust; 2 Public Advisor with lived experience, Early Break; 3 NIHR ARC NWC* Research Associate, University of Liverpool

**Keywords:** parental mental health, children’s social services, critical appraisal, systematic review, trauma-focused care

## Abstract

**Background:**

Children of parents with a diagnosed mental illness are four times more likely to be removed by social services worsening parental mental health and introducing further trauma. A previous review synthesised the evidence on the views and experiences of parents with mental health problems (MHPs) and social care practitioners regarding the support provided.

**Aim:**

This commentary aims to critically appraise this review and expand upon its findings in the context of current evidence and practice.

**Methods:**

The quality of the review was assessed using the Joanna Briggs Institute Critical Appraisal Checklist.

**Findings:**

Despite potential biases in the review and some methodological issues in the included studies, the findings reveal important challenges in supporting parents with MHPs.

**Conclusion:**

Mutually trusting relationships between families and professionals are key in facilitating engagement. A strengths-based holistic approach would yield more positive outcomes for all. Adequate central government funding for local authorities is essential to deliver the care required.

## Introduction

Poor maternal mental health can adversely affect early infant caregiving, potentially resulting in neglect, maltreatment, and the perpetuation of intergenerational trauma (Lopes et al., 2021). It has been associated with both maternal and infant mortality and morbidity, in addition to high health and social care costs (Howard & Kaliefah, 2019). Children of parents with a diagnosed mental illness are four times more likely to be removed and the risk of removal further increases when multiple socioeconomic disadvantages present (Nevriana et al, 2024).

Having a child removed by social services exacerbates mothers’ mental health difficulties (Broadhurst & Mason, 2020). These mental health challenges along with the stigma mothers experience often create further trauma affecting future pregnancies and potentially leading to future care proceedings (Morriss, 2018). Relationships between parents and services are damaged through the process, creating mutual mistrust, which in turn can lead to parental disengagement and disguised compliance, stress and burnout in social care workers, and ultimately an increased risk of negative outcomes for children (Mason et al 2020).

Systematic reviews have either considered parents’ mental health needs (Radley et al., 2022) or child protection interventions (van der Put et al., 2018). Combining these two aspects is important both in research and practice to better meet the needs of parents with mental health problems who have been involved with children’s social services (McPherson et al., 2018). The review by Bacon et al (2023) aimed at examining and synthesising the evidence available on the views and experiences of parents with mental health problems as well as social care practitioners in relation to the support and intervention provided to families.

### Aim of commentary

This commentary aims to critically appraise the methods used within the review by Bacon et al (2023) and expand upon the findings in the context of social care practice.

#### Commentary approach

This critical review utilises a RaCES (Rapid Conversion of Evidence Summaries) project methodology. The commentary is a collaborative work between academics, health and care professionals, and people with lived experience converting systematic reviews into evidence summaries to build research capacity in health and care professionals, develop professional networks, and inform practice using the latest scientific evidence. The Joanna Briggs Institute Critical Appraisal Tool (Aromataris et al, 2015) for systematic reviews and research syntheses was used to evaluate the review.

## Methods of Bacon et al (2023)

This protocol registered systematic review used multiple and relevant databases, including grey literature to carry out the literature search. The search was limited to reports published from 2000 onwards to ensure relevance to current service provision, although the date of search was not reported. Two reviewers independently screened 10% of the titles, abstracts, and full texts of the studies and disagreements were discussed in the wider team. Included studies needed to examine parents’ or practitioners’ views on support for parents with mental health difficulties and social services involvement, using qualitative or mixed methods (with separate qualitative data), addressing experiences of professional support or interventions, and be peer-reviewed and published in English.

The Critical Appraisal Skills Programme (CASP) qualitative checklist (2018) was used to assess the quality of included studies, with three additional areas: intersectionality, positionality, and service user involvement. Ten percent of the studies were assessed independently by two reviewers and discrepancies were resolved through discussions. Data was analysed using thematic synthesis (Thomas & Harden, 2008). Ten percent of the data was independently coded by two authors who then compared coding frames and developed a shared understanding of the data. Discussions with a lived experience advisory group informed data analysis and together with clinicians they assisted in the interpretation of findings.

## Results by Bacon et al (2023)

The search identified 11,334 papers. After removing duplicates and screening titles and abstracts, 242 reports were assessed for eligibility through full-text screening. The review included 41 studies (39 peer reviewed, two reports of charities). The main characteristics of included studies can be found in Table 1.

The main quality issues identified by the CASP were around a lack of exploration of relationships between researcher and participants and consideration of ethical issues. Data analysis was not deemed sufficiently rigorous in 19 studies as it was unclear how analytical themes were derived from the data. Moreover, in terms of the additional areas of intersectionality, involvement of service users, and positionality that were added to the assessment, studies lacked discussion.

Four main themes with several sub-themes reflecting both the views of the practitioners and the parents were identified.

### Theme 1: “A downward spiral of service intervention”

According to this theme, service interventions often escalate problems rather than solve them. Parents feel judged and stigmatised. While they need support, they are reluctant to seek it and find it challenging to receive it as it tends to worsen mental health problems and suicidality due to pressures, custody disputes, and intrusive home visits. Parents feel (re)traumatised and therefore try and avoid engagement, which is viewed as non-compliance. Additionally, mental health treatment interferes with parenting (e.g. due to the side effects of medication). Practitioners feel that in order to protect children, removal is sometimes necessary but at the same time they are acutely aware of the negative impact of this on the parents.

### Theme 2: “Working with Parents, Not against them”

The importance of rapport between parents and practitioners was the focus of this theme. Many of the relationships are characterised by mistrust due to parents’ fear of losing custody of the child leading to engagement difficulties. Mental health clinicians are reported to be more likely to make an effort to build positive relationships, which result in better outcomes. While parental trauma due to abuse in childhood and/or adulthood is clear and often recognised, parents feel that this does not necessarily translate into appropriate, empathic, and blame-free trauma-informed care. Additionally, deficit-focused care where recovery is considered symptom management (by professionals) diminishes parents’ confidence in their parenting ability, strengths-focused approaches are seen to have the potential to motivate parents and improve engagement who think of recovery in terms of aspects, such as parenting role and connections to others. Finally, to facilitate engagement, professionals should be transparent about their expectations, communicate with parents regularly, clearly, and without using jargon, and work in collaboration with parents towards shared decision making. Parents viewed these as key components of good support.

### Theme 3. “Support wanted versus support provided”

This theme identified a discrepancy between the perspectives of parents who wanted flexible support and professionals who worked in a rigid way by the books. Parents wish to have their parenting role and the impact of losing custody of a child acknowledged and incorporated into their mental health treatment through non-judgemental parenting and emotional support. Parents also feel that that they need financial help to achieve valued outcomes, such as retaining custody of their child, but this is rarely offered. In addition to parenting and financial support, parents felt that appropriate mental health support that involved not only diagnosis/labelling and medical treatment, but also considered the person behind the diagnosis was essential to reduce negative assumptions and judgement and avoid pathologisation of parenting difficulties. What parents find beneficial, although seldom provided, is the use of psychological therapeutic approaches, particularly in a group setting where they can utilise peer support which helps normalise their experiences. Finally, the availability of a strong social support network (e.g. family, friends, church) was perceived to have benefits by parents in reducing isolation and assisting with crisis. Yet, the role of the family, including the role and responsibilities of fathers, has been neglected by both support services and research.

### 4 “Constrained by Service Rigidity”

Inadequate resources, rigid processes, and service inaccessibility were reported by professionals as factors restricting professional practice. Better integrated service provision was found to be linked to better outcomes (e.g. reduced incidents of child removal). However, a lack of integration of and collaboration between services was found resulting in fragmented support and professionals’ inability to take a holistic view of their clients that considers the complexities of each case. For example, while social services seem to have a sole focus on risks to children, mental health clinicians focus on the risks to parents and feel that parenting-related issues are beyond their role. Professionals felt that this makes it difficult to make decisions that serve parents and children well. Findings also showed that service separation and a lack of collaboration were more prevalent in England compared to European countries. Both professionals and parents agreed that mental health support was crisis-driven without preventative action, which leads not only to challenges for parents, such as losing custody of their child(ren), but poorer wellbeing and burnout for professionals. Services provided lack of cultural sensitivity. Access to support is challenging in terms of practicalities (e.g. shortage of childcare, travel distance), as well as lack of flexibility about eligibility for mental health support.

## Commentary

### Critical appraisal of the review by Bacon et al (2023)

Using the JBI tool (Aromataris et al., 2015) for systematic reviews (Table 2), eight out of the 11 criteria were judged satisfactory for this review. The title, abstract and full text screening, data extraction, and quality assessment of the included studies were not performed by two researchers independently potentially leading to selection bias, inaccurate estimation of risk of bias, and higher likelihood of errors during data extraction. While reference was made to searching grey literature, publication bias was not considered or discussed. Selective dissemination of studies in qualitative research may lead to missing important findings, which has the potential to skew our understanding of the phenomena at hand (Booth et al., 2018). The potential biases described above in addition to the methodological issues of the included studies as identified by the review authors reduce our confidence in the findings. Nevertheless, the findings of this review reveal important challenges in terms of supporting parents with mental health problems.

According to the findings, while parents need support, interventions often trigger a downward spiral as parents feel that services are working against them, retraumatising parents and worsening their mental health leading to perceived disengagement. It is important to recognise that parental mental health issues are often a result of trauma which will have an impact on the parents’ response to interventions (Suomi et al., 2023). There is a perception that first encounter between social workers and parents can be emotionally highly charged because parents are driven by fear, anger, and mistrust. This, alongside inadequate communication and deficit-focused care, makes it challenging to establish a mutually trusting relationship, which would be necessary for positive parental engagement and change (Broadhurst & Mason, 2020).

To successfully intervene and create positive outcomes for the entire family, the optimal approach would be founded in trauma-informed care (Bunting et al., 2019). Using a bottom-up approach, where peers with lived experience are involved in the engagement, assessment, and support of parents could facilitate better communication and stronger relationships (Wessells, 2015). A collaborative approach with shared decision-making can enable the reduction of stigma, forming connections with parents who otherwise struggle to engage with services, and have the potential to instil hope and genuine participation (Parkinson, 2021).

Positive trusting relationships between parents and social care enables early identification and management of family needs, which may positively influence child outcomes (Narayan et al., 2021). Taking a more preventative approach and engaging families and communities have been found effective in protecting children from harm, improving family life, reducing the need for child protection services involvement, combating the cycle of intergenerational trauma and abuse, and supporting children to develop strengths and skills that prepares them for adulthood (Narayan et al., 2021). This in turn can improve children’s long-term outcomes (Bethell et al., 2017). Additionally, a non-judgemental, whole-family, strengths-based approach has the potential to boost confidence in parenting which can improve the experience for the child and the parent, as well as the practitioner-parent relationship (Waters & Sun, 2016; Murphy et al, 2013).

Parents described their need for parental, mental health, and financial support, and they called for the inclusion of their social support network. Acknowledging the parent’s emotional responses and needs as valid and providing therapeutic mental health support alongside the child protection process may be the most helpful for the individual (Levenson 2017). Different professionals working together in an integrated approach, would more effectively meet the complex needs of families and reduce the negative outcomes for both parent and child (Jahans-Baynton & Grealish, 2021). For example, employing adult specialist mental health practitioners within children’s social care teams has been shown to achieve a 30% reduction in children becoming looked after and mental health crisis calls (Rodger et al, 2020). Finally, financial hardship often goes hand in hand with mental health issues (Kiely et al., 2015) and child neglect (Gupta, 2017). Therefore, alongside parental and mental health support, economic programmes would be useful to provide financial aid to help parents get back on their feet.

However, practitioners’ narratives reveal the challenging nature of meeting the above-described needs when working within the constraints of inadequate resources, rigid processes, and inaccessible and segregated services. These are the perceived barriers to providing holistic care and taking a preventative approach which is perceived to lead to parental disengagement and negative outcomes for all. Due to workload pressures, including high caseloads, training and supervision for social workers have become low priority (Rothwell et al., 2021) despite clear requirements by the British Association of Social Workers (BASW, 2021). This has led to social workers being ill-equipped to deal with the variety of complexities presented in practice resulting in high levels of burnout (Davidson, 2024). However, due to a reduction in central government spending (Marmot, 2020), local authorities in the UK lack the funds to source economic programmes for families, and to invest in child protection services to increase resources, improve working conditions for staff, create more effective processes, and ultimately enable systemic change. Furthermore, cuts were higher in more disadvantaged local authorities, hence their spending power greatly decreased (Atkins & Hoddinott, 2020). There is need for support from the central government to enable local authorities to deliver the care that is required, and resource allocation and implementation of policies should be proportionate to need (Marmot, 2020).

#### Recommendations for future research

Future reviews could consider extracting first-order constructs to minimize the risk of losing the essence of the original studies (Noyes et al., 2018). Additionally, dissemination (publication) bias and its potential impact on the findings in the context of qualitative evidence syntheses, whilst not well understood, should be considered when assessing how much confidence we have in findings from qualitative evidence syntheses (Booth et al., 2018).

As discussed by the review authors, most of the perspectives were those of professionals. More primary research is needed that investigates the views of parents, and in particularly fathers’ perspectives, roles, and responsibilities. Primary research should be clearer about population characteristics to enable the exploration of differences in experiences for people with different socioeconomic backgrounds, and social and cultural characteristics. Despite the complex interaction between financial deprivation, ethnicity, and child protection interventions (Webb et al., 2020), the current evidence does not take an intersectional approach that could reveal any inequities, which should be remedied in future work.

## Conclusion

While parents with mental health problems need support, they are reluctant to engage with children’s social services due to the stress it causes. Building strong relationships, utilising peer support, providing trauma-informed care, and taking a non-judgemental, whole-family, strengths-based approach will enable more successful interventions and better outcomes for both families and professionals. However, the help of central government is needed to enable local authorities to deliver the care that is required. Further research is needed to explore differences in experiences of different populations and to reduce health inequalities.

## CPD reflective questions

1. How could this review be carried out to yield more robust results?
2. How do you think the participation of more parents in research would potentially change or expand the findings?
3. What could you do within your practice to create better relationships with families?

## Figures and Tables

**Table 1:** Main characteristics of included studies

**Study date**	2001-2014 – 18 studies
	2015-2022 – 23 studies
**Country of origin**	United Kingdom –13 studies
	United States – 10 studies
	Australia – 10 studies
	Canada – 3 studies
	Sweden – 2 studies
	New Zealand – 2 studies
	Japan - 1 study
**Number of participants**	Parents – N=337
	Professionals – N=1370
**Population**	Parents and professionals – 10 studies
	Parents only – 15 studies
	Professionals only – 16 studies
**Type of professionals**	Social workers, family support workers, case managers, child protection workers, psychologists, psychiatrists, nurses, housing shelter workers, general practitioners, solicitors
**Parental mental health** **problems**	Depression, anxiety, psychosis, personality disorders, substance/alcohol use

**Table 2:** Critical appraisal using the Joanna Briggs Institute Critical Appraisal Checklist (Aromataris et al, 2015) for systematic reviews and research syntheses

JBI critical appraisal checklist items	Responses
1. Is the review question clearly and explicitly stated?	**Yes**
2. Were the inclusion criteria appropriate for the review question?	**Yes**
3. Was the search strategy appropriate?	**Yes**
4. Were the sources and resources used to search for studies adequate?	**Yes**
5. Were the criteria for appraising studies appropriate?	**Yes**
6. Was critical appraisal conducted by two or more reviewers independently?	**No** – only 10% of the studies were appraised independently by two researchers and discrepancies were resolved through further discussion.
7. Were there methods to minimize errors in data extraction?	**No** – Information was not reported on who performed data extraction.
8. Were the methods used to combine studies appropriate?	**Yes**
9. Was the likelihood of publication bias assessed?	**No** – publication bias was not considered and discussed although, the authors searched the grey literature, which can potentially reduce publication bias.
10. Were recommendations for policy and/or practice supported by the reported data?	**Yes**
11. Were the specific directives for new research appropriate?	**Yes**

## Key Points

- Despite potential biases in the review and some methodological issues in the included studies, the findings reveal important challenges in supporting parents with mental health problems.
- While difficult, it is key to develop mutually trusting relationships with families and take a strengths-based, holistic, and, if possible, preventative approach to support.
- Provision of effective support is difficult to achieve due to inadequate resources, rigid processes, service inaccessibility and segregation, leading to parental disengagement and negative outcomes for parents, children, and professionals.
- Central government should appropriately fund local authorities to enable them to support social services to deliver the care that is required.
